# Round the Hospitals

**Published:** 1921-02-05

**Authors:** 


### ROUND THE HOSPITALS.

The General Nursing Council for England and Wales sat for three hours on Wednesday, February 2, and discussed a variety of important and interesting matters. The Registration Rules, which were sent to the Minister of Health so long ago as September last, are still awaiting his sanction, and meanwhile it appears that certain alterations in those rules are being put forward from time to time. At Wednesday's meeting, for instance, two important resolutions were passed involving alterations to the Rules. They deal with the recognition, for the purposes of the Registers, so far as existing nurses are concerned, of the training in a general hospital for children, together with certain training in a general hospital or Poor-Law infirmary approved by the Council. These alterations, if sanctioned, will help the position of the sick children's nurse. It was stated that the Minister of Health had sent no reply to the request that he would bring in a bill to regulate nurses' hours, made as directed by the Council at its last meeting, and Mr. Christian's resolution in effect to rescind that request and to recommend that nurses should come within the scope of the Ministry of Labour's Hours of Employment Bill was defeated. We shall publish a full report of the meeting in our next issue. The Council realises the necessity for propaganda in order to enlighten the nurse as to the scope of its powers and functions. There are even now nurses and members of the medical profession who appear to be unaware of the establishment of the General Nursing Council, and to have but the haziest idea of the provisions of the Nurses' Registration Act. A vigorous scheme for countering this apathy and removing the ignorance is outlined in the report of the Registration Committee, which was adopted by the Council at Wednesday's meeting. The recommendation of this Committee, that the opinion of all nurses should be sought on the questions of the uniform and badge, was also adopted. Members of the profession will thus be enabled to express their views and wishes on this vexed question, and we hope they will take full advantage of it.

The General Nursing Council for Scotland has agreed to delete the draft rule which provided for the Council accepting special evidence of adequate knowledge and experience of nursing in cases where existing nurses applying for admission to the General Register have no hospital training. The difficulty of obtaining and weighing such special evidence has been deemed insuperable. Everyone knows the kind of evidence which would be brought, signed by ministers of religion and easily satisfied relatives of patients. Hospital training, varying in length it is true, has been held indispensable to the making of a trained nurse for nearly half a century, and to put women on the Register who have never been inside a ward would irretrievably lower the Register for a generation to come. The Scottish decision to form a separate supplement for nurses trained in the care of mental defectives is popular with workers in these institutions and the authorities controlling them. The course of training differs in many particulars from that required for the nursing of the insane, and it is desired that the two groups of nurses shall be regarded as definitely distinct. The vexed question relating to fever nurses is still unsettled.

It would appear, from the very poor attendance at the excellent professional lectures given under the auspices of the London Centre of the College, that there is but a small demand among nurses in London for post-graduate instruction. We may be mistaken in this surmise, but it has often been brought to our notice how difficult it is in London to secure a good audience for discourses from men and women in the forefront of their profession. It is partly due to the influence of the place. Every leisure hour finds the Londoner with half a dozen attractions tugging him in different directions. But it may also be due to recoil from any sustained effort of attention which does not "count." A short set of lectures, with an examination at the end, crowned with a certificate, would attract the earnest student, and such courses would be of permanent

**ROUND THE HOSPITALS**—(*continued*).

value. We commend this suggestion to the authorities, and meanwhile we call our readers' attention to the lecture by Sir Robert Armstrong-Jones on "The Relation between Mental Nursing and Hospital Nursing," full particulars of which appear on page vi. The subject is one of particular interest to all nurses, and they should not fail to hear what so noted an authority as Sir Robert Armstrong-Jones has to say.

THE death is reported by cable of Miss Beatrice Isabel Jones, C.B.E., R.R.C. matron-in-chief in Mesopotamia, who passed away at Baghdad on the 15th ult. Miss Jones, who was trained at St. Bartholomew's Hospital in 1891-94, had rendered notable service to the military forces, whilst her work in civil hospitals before she entered the Army Nursing Service was of no less importance. After her training she did district work in Liverpool, returning to her training-school as assistant home sister and night superintendent, going thence to the new Birmingham Infirmary as assistant matron and afterwards to Victoria Park Hospital as matron. She served in South Africa in the Boer War as a sister, and was appointed matron Q.A.I.M.N.S. in 1903. She held important charges in various military centres at home and in Egypt, and during the late war held the post of acting Principal Matron in Mesopotamia, where she rendered exceptionally valuable service. She returned to England in 1920, and was seconded from the Q.A.I.M.N.S. for service under the Civil Administration in Mesopotamia. Hers was a life of strenuous and devoted work which has ended as it had been lived—in harness. In addition to the distinctions mentioned after Miss Jones's name, she was twice mentioned in despatches, and was awarded a Bar to the Royal Red Cross and the Florence Nightingale International Medal.

FEW people have any idea of the extent to which nurses are aiding the work of bringing this region of the East to blossom like the rose. The civil hospitals in Mesopotamia with a trained nursing staff include the new General Male Hospital, the Civil Female Hospital, the Venereal Hospital for Women, and the Serai Nursing Home for Officers and Ladies, all at Baghdad; the Civil Isolation Hospital and the Civil Nursing Home at Basra; and small hospitals at Diwaniyah, Hillah, Nasariyeh, Kut, Kerkuk, Mosul and Amarah. The nursing of all these, as well as of dispensaries at Baghdad and elsewhere, was under the chief control of Miss Beatrice Jones, who had a trained staff of upwards of twenty-five sisters. It was Miss Jones's aim to organise a system to train the natives in the principles of hygiene, first-aid, and the care of eyes, and there is no doubt that, had she lived, she would have accomplished her purpose, for not only was she an indefatigable worker herself, but she possessed the power of inspiring those under her to put their best and their utmost into the work in hand. She went out to Mesopotamia last year with high hopes, looking forward keenly to the task before her, for, as she said to a friend just before her departure, she loved every grain of sand of the country.

AT a meeting of the Central Midwives Board for Scotland, held in Edinburgh, Sir J. Halliday Croom in the chair, the Viscountess Novar was introduced to the meeting and took her seat as a member of the Board. On concluding the ordinary business, Sir Halliday Croom, in a valedictory address on his retiral from the Board, thanked the members for the loyal support he had received as Chairman, and referred to the pleasant and harmonious way in which the work had been carried out. On the motion of Dr. A. Campbell Munro, seconded by Sir Archibald Buchan-Hepburn, a hearty vote of thanks was accorded to Sir Halliday Croom for the efficient manner in which he had discharged the important duties of Chairman of the Board since its formation during a period of five years. The Board also expressed its appreciation of the able and valuable services rendered by Mr. D. L. Eadie, the Secretary. The constitution of the new Board appears on p. 438.

SINCE 1910 night sisters have been appointed in all the institutions for infectious diseases under the Metropolitan Asylums Board under a grandmotherly stipulation that the maximum period for which anyone may act continuously in this capacity should be one year. The excellent intention was, no doubt, to prevent the health of any sister from deteriorating under prolonged night work. Strange as it may seem to outsiders, however, night work is very popular with certain women; its strain is usually felt during the first month or two only of appointment, and the change to day work is positively disliked when the habit of reversing the hours of sleep and occupation has been acquired. Accordingly the Board has now extended the maximum term of night service to three years, which may be again extended at the discretion of the sub-committee dealing with the nursing staff. This is a very sensible arrangement, likely to be highly popular with those whom it concerns.

WE understand that the report of the special committee appointed by the Islington Board of Guardians to inquire into the adverse comments of Miss Wamsley, inspector, of the Ministry of Health, on the nursing at the Islington Infirmary is to be brought before the Guardians at their meeting on February 11. Meanwhile the Poor-law Infirmary Matrons' Association, at a largely attended quarterly meeting of the Association on January 29, passed a unanimous resolution extending the meeting's very real sympathy to the matron and nursing staff of the infirmary. The resolution continues that by this means a very misleading and unfair impression was conveyed to the public in connection with an infirmary which is amongst the foremost of Poor-law training-schools for nurses, the nursing staff of which have always held an honoured position in the nursing world.

